# Role of inflammasomes in cancer immunity: mechanisms and therapeutic potential

**DOI:** 10.1186/s13046-025-03366-y

**Published:** 2025-03-29

**Authors:** Vivek Singh, Saba Ubaid, Mohammad Kashif, Tanvi Singh, Gaurav Singh, Roma Pahwa, Anand Singh

**Affiliations:** 1 Thoracic Surgery Branch, Center for Cancer Research, National Cancer Institute, National Institutes of Health, Bethesda, MD 20892 USA; 2 Department of Biochemistry, King George’S Medical University (KGMU), U.P, Lucknow, 226003 India; 3 Laboratory of Malaria and Vector Research, National Institute of Allergy and Infectious Diseases, National Institutes of Health, Rockville, MD USA; 4 Urologic Oncology Branch, Center for Cancer Research, National Cancer Institute, National Institutes of Health, Bethesda, MD 20892 USA

**Keywords:** Inflammasomes, Cancer immunity, NLRP3 inflammasome, Innate and adaptive immunity, Tumor microenvironment, Caspase-1, Pyroptosis, Inflammasome-targeted therapy

## Abstract

Inflammasomes are multi-protein complexes that detect pathogenic and damage-associated molecular patterns, activating caspase-1, pyroptosis, and the maturation of pro-inflammatory cytokines such as IL-1β and IL-18Within the tumor microenvironment, inflammasomes like NLRP3 play critical roles in cancer initiation, promotion, and progression. Their activation influences the crosstalk between innate and adaptive immunity by modulating immune cell recruitment, cytokine secretion, and T-cell differentiation. While inflammasomes can contribute to tumor growth and metastasis through chronic inflammation, their components also present novel therapeutic targets. Several inhibitors targeting inflammasome components- such as sensor proteins (e.g., NLRP3, AIM2), adaptor proteins (e.g., ASC), caspase-1, and downstream cytokines- are being explored to modulate inflammasome activity. These therapeutic strategies aim to modulate inflammasome activity to enhance anti-tumor immune responses and improve clinical outcomes. Understanding the role of inflammasomes in cancer immunity is crucial for developing interventions that effectively bridge innate and adaptive immune responses for better therapeutic outcomes.

## Background

Inflammasomes, large multi-protein complexes that form in response to endogenous danger signals within the cytosol, are key players in triggering inflammatory immune responses. Their formation activates caspase-1, a critical inflammatory protease, leading to pyroptosis and the conversion of pro-inflammatory cytokines, such as pro-IL-1β and pro-IL-18, into their active forms [1]. The complex comprises a Nod-like receptor (NLR), the adaptor protein ASC, and caspase-1. While the NLRP3 inflammasome plays a crucial role in tumor pathogenesis, its role in cancer development and progression remains controversial due to conflicting findings [2]. The inflammatory tumor microenvironment is intricately involved in all stages of cancer, from initiation to promotion and progression. Systemic inflammation in this environment has become a significant prognostic indicator in cancer patients [3, 4]. Inflammasomes play a central role in this process by detecting pathogen-associated molecular patterns (PAMPs) and damage-associated molecular patterns (DAMPs), which signal the recruitment of immune cells to sites of infection or injury [5, 6], dysregulation of inflammasome expression or activation linked to various inflammatory disorders. Recent evidence highlights the role of inflammasomes in cancer development and progression, with particular emphasis on the downstream effector cytokine IL-1β [7, 8]. Emerging research has shown that inflammasome activation can be triggered by oncogenic stress and metabolic disturbances, although its full role in cancer biology remains unclear. NLRP3 has emerged as a critical factor influencing tumor progression, functioning through both pro- and anti-tumorigenic pathways [9, 10]. Understanding these mechanisms is crucial as it can potentially modulate inflammasomes for therapeutic purposes, including tumor growth inhibition and metastasis control. This review aims to inform the audience about the intricate role of NLRP3 in cancer by summarizing findings across various tissue contexts and cancer hallmarks, focusing on evaluating its potential as a therapeutic target.

**Fig. 1:**
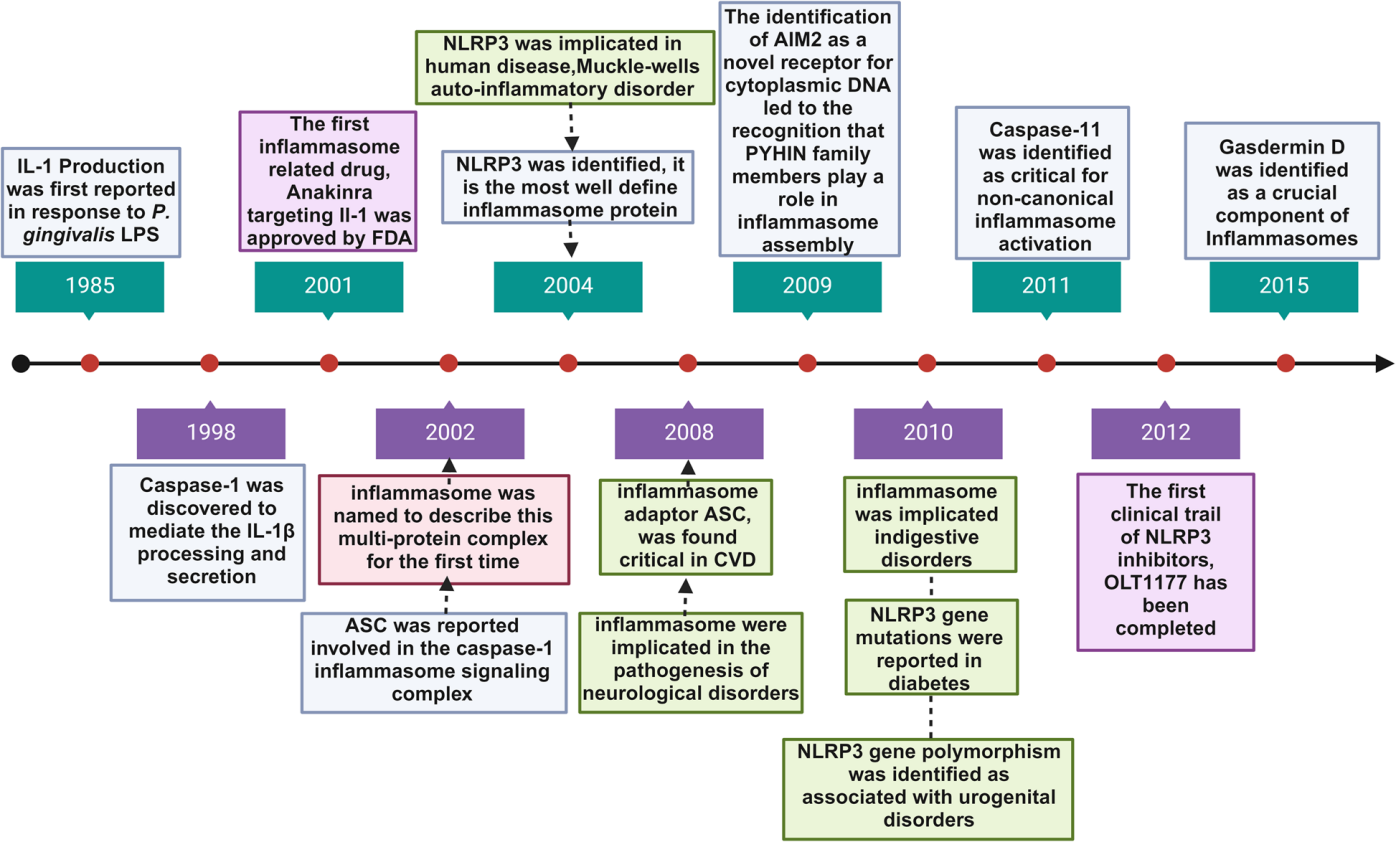
An overview of key milestones in inflammasome research and their applications. Blue boxes indicate discoveries of key components of different inflammasomes; the pink box marks the origin of the term "inflammasome"; green boxes show associations between inflammasomes and various human diseases; and purple boxes represent representative clinical applications of inflammasome-related modulators

Inflammasomes have emerged as potential targets for disease-modifying therapies, with several clinical trials underway to investigate their therapeutic potential (Fig. 1). These ongoing trials could lead to new therapeutic regimens for various inflammatory disorders and cancers [11–13]. The innate immune pathway has gained traction in cancer therapy due to its role in different cell types, including immune, tumor, and stromal cells. Preclinical studies have shown that targeting this pathway with specific agonists can remodel the tumor microenvironment and improve the prognosis [14]. However, clinical success is still limited, as activation of this pathway can also promote tumor progression, depending on factors such as tumor stage, pathway status, and the specific organ or tissue involved. A deeper understanding of the innate immune pathway within the tumor microenvironment could enhance its anti-tumor potential and improve clinical outcomes [15]. Moreover, inflammasomes, as key players in regulating innate and adaptive immunity, provide a fascinating bridge between these two arms of the immune system. This review explores how inflammasomes link these two arms, mainly focusing on their role in immune responses and their interaction with metabolic signals. These insights provide valuable opportunities for identifying new therapeutic targets [16]. While adaptive immunity helps to control or prevent cancer growth, innate immunity, and inflammation often contribute to tumor initiation and progression. Despite significant progress in developing cancer immunotherapies, targeting cancer-related inflammation remains challenging. Anti-inflammatory therapies promise to prevent or delay cancer onset and enhance traditional treatments and immunotherapies [17]. This review highlights recent research and clinical developments that support anti-inflammatory strategies in treating solid tumors, emphasizing the importance of understanding the underlying mechanisms for designing more effective treatment combinations [18].

### Inflammasome

**Fig. 2:**
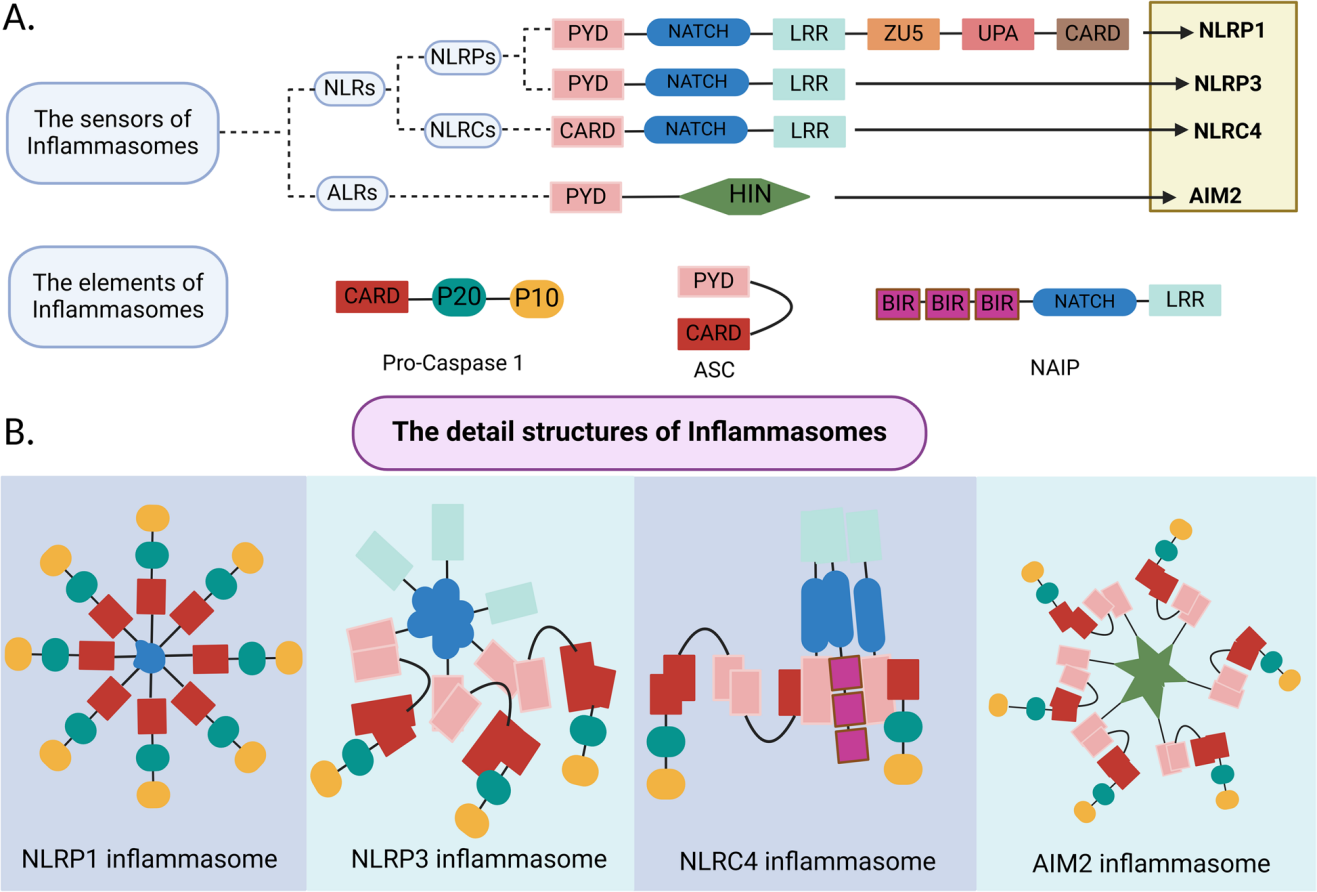
Representative structures of inflammasome sensor proteins and their complex assemblies.** A** This figure illustrates the representative structures of inflammasome sensor proteins, including NLRPs (such as NLRP1 and NLRP3), NLRCs (such as NLRC4), and ALRs (such as AIM2). **B** It also highlights the representative structures of various inflammasomes, such as the NLRP1, NLRP3, NLRC4, and AIM2 inflammasomes. Key terms include: NLRs (Nucleotide-binding domain, leucine-rich repeat-containing proteins), NLRPs (A subset of NLRs with an N-terminal pyrin domain (PYD)), NLRCs (NLRs with a caspase activation and recruitment domain (CARD)), ALRs (AIM2-like receptors, such as absent in melanoma 2), NAIP (NLR apoptosis inhibitory protein)

**Fig. 3:**
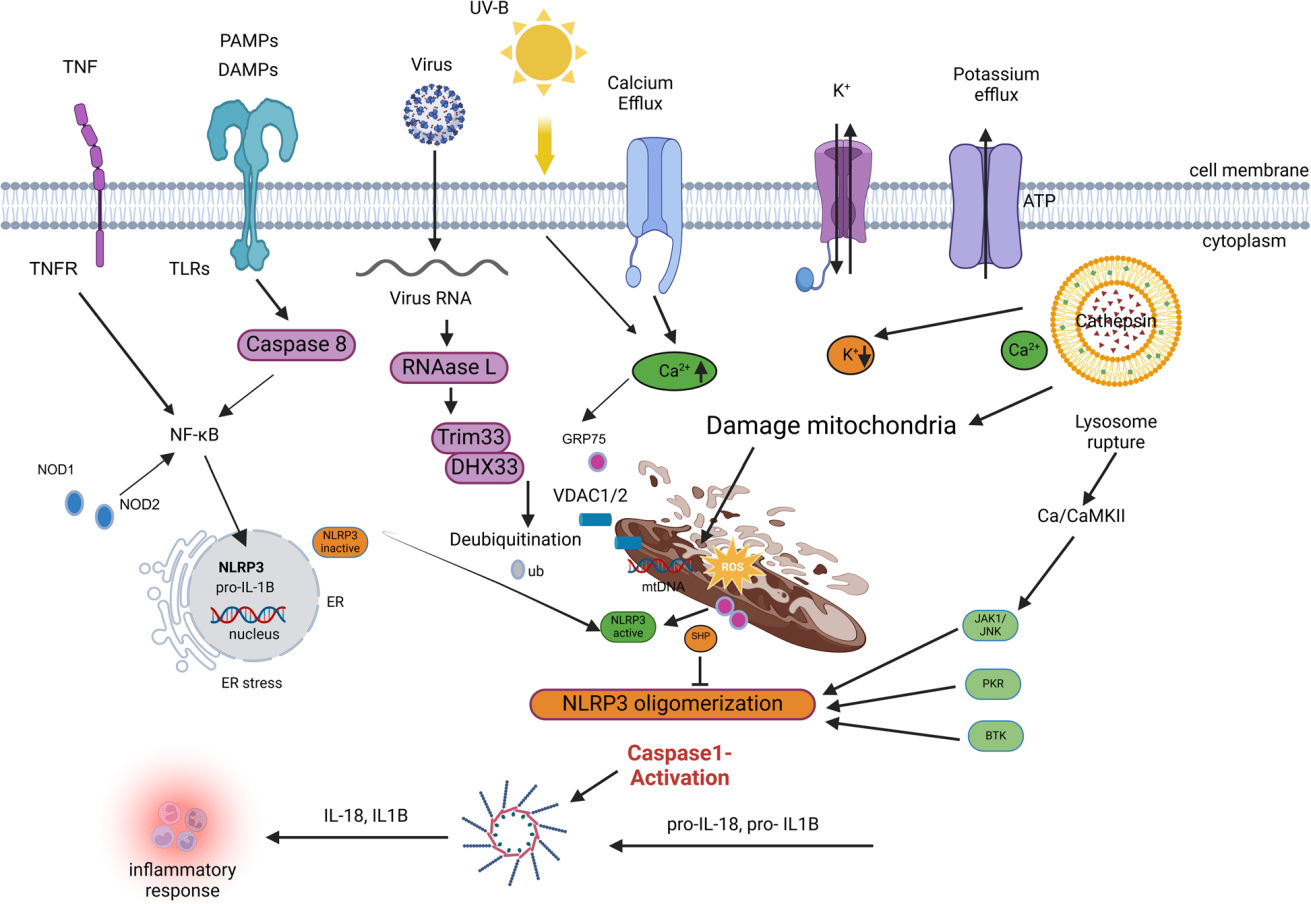
Mechanism of Activation and Signaling Pathways of the NLRP3 Inflammasome. This figure illustrates the activation and signaling mechanisms of the NLRP3 inflammasome, featuring key components such as Bruton's tyrosine kinase (BTK), calcium/calmodulin-dependent protein kinase II (CaMKII), and protein kinase R (PKR). It highlights how damage-associated molecular patterns (DAMPs) and pathogen-associated molecular patterns (PAMPs) interact with nucleotide-binding oligomerization domain (NOD)-like receptors like NLRP3 (NOD-like receptor family pyrin domain-containing 3). Other important elements include DEAH-box helicase 33 (DHX33), the endoplasmic reticulum (ER), interleukins (IL), Janus kinase 1 (JAK1), lipopolysaccharide (LPS), the mitochondrial calcium uniporter (MCU), nuclear factor kappa B (NF-κB), the small heterodimer partner (SHP), tumor necrosis factor (TNF), tripartite motif-containing protein 33 (TRIM33), and voltage-dependent anion channels 1 and 2 (VDAC1/2)

The term "inflammasome" was coined by Tschopp and colleagues in 2002 to describe a protein complex responsible for activating inflammatory caspases [19]. This collaborative effort has significantly advanced our understanding of the immune system. Inflammasomes are large molecular complexes that become activated in response to various pathogen infections or cellular stresses, rapidly producing pro-inflammatory cytokines. These cytokines play a critical role in recruiting innate immune cells to fight off invading pathogens [20, 21]. Dysregulation of inflammasome activity has been associated with tumor development [22]. Inflammasomes are broadly categorized into "classical" and "non-classical" inflammasomes based on the specific caspase enzymes they activate [23]. Classical inflammasomes comprise several proteins, including pattern recognition receptors (PRRs), the adaptor protein ASC, and pro-cysteine-requiring aspartate protease-1 (pro-caspase-1). Interestingly, some PRRs are also expressed in adaptive immune cells, which regulate antigen presentation [24]. PRRs involved in innate immunity are classified into two main types: transmembrane proteins, such as Toll-like receptors (TLRs) and C-type lectin receptors (CLRs), and cytoplasmic proteins, including RIG-I-like receptors (RLRs) and Nod-like receptors (NLRs) [25]. The discovery of the first inflammasome-related PRR in 2002 led to the identification of multiple inflammasomes, including NLRP1, NLRP2, NLRP3, absent in melanoma 2 (AIM2), and NLRC4 [26, 27], as shown in Fig. 2. Among these, the NLRP3 inflammasome, also known as pyrin domain-containing protein 3, has been extensively studied for its role in inflammation [28]. NLRs are cytoplasmic PRRs, and their activation requires two signals for optimal function. The first signal, or priming, is initiated by external stimuli like tumor necrosis factor (TNF), IL-1β, or pathogen-associated molecular patterns (PAMPs), which enhance the expression of inflammasome components and pro-inflammatory cytokines. The second signal, typically provided by PAMPs or damage-associated molecular patterns (DAMPs), results in the oligomerization and assembly of inflammasome components [29]. The formation of inflammasomes involves oligomerizing receptor proteins such as NLRP1, NLRP3, NLRC4, AIM2, and pyrin. PAMPs are recognized by PRRs, which are essential for detecting pathogenic threats or cellular damage. DAMPs, including extracellular ATP, ion flux changes, lysosomal damage, mitochondrial reactive oxygen species (mtROS), and oxidized mitochondrial DNA (ox-mtDNA), also activate the NLRP3 inflammasome [30, 31]. Upon PRR activation, classical inflammasomes bind to ASC via domains such as the caspase recruitment domain (CARD) or pyrin domain (PYD), forming oligomeric structures that recruit and activate pro-caspase-1. Activated caspase-1 cleaves pro-interleukin-1β (pro-IL-1β) and pro-interleukin-18 (pro-IL-18) into their mature, active forms. These cytokines facilitate immune responses by recruiting inflammatory and immune cells to the site of infection, thereby contributing to innate immunity [10]. Active IL-1β functions as a potent pro-inflammatory cytokine, attracting immune cells to the affected site, while active IL-18 promotes interferon-γ (IFN-γ) production, which enhances the activity of natural killer (NK) cells and T cells [20, 32]. Caspase-1 also cleaves Gasdermin D (GSDMD), causing pore formation and lysis of the cell membrane, which leads to a distinct form of inflammatory cell death known as pyroptosis [33]. In contrast, non-classical inflammasomes primarily activate caspases such as Caspase-3, 4, 5, 7, 8, and 11 rather than caspase-1 [23], as illustrated in Fig. 3.

### The role of inflammasomes in health and disease

Dysregulated inflammasome activity has been associated with the onset and progression of a wide range of diseases, including diabetes, cancer, and various cardiovascular and neurodegenerative disorders. Consequently, recent studies have increasingly focused on elucidating the mechanisms underlying inflammasome assembly and activation and exploring their potential as therapeutic [34–36]. Importantly, several clinical trials are currently underway to assess the efficacy of different therapies that modulate inflammasome activity, providing a real-time update on the state of research. Understanding the contributions of various inflammasomes to disease pathogenesis could significantly impact the development of novel therapeutic strategies [37, 38]. Notably, Inflammation is closely linked with the hallmarks of cancer. NLRP3 plays a dual role in cancer, contributing to tumor-promoting inflammation and immune evasion while influencing immune surveillance. In cervical and prostate cancers, NLRP3 is activated by pathogens and oncogenic stress [2, 38, 39]. In breast cancer, it enhances sphingosine-1-phosphate (S1P) signaling in tumor-associated macrophages (TAMs) and promotes metastasis by recruiting γ/δ T cells [40, 41]. In colorectal cancer (CRC), liver metastasis, fibrosarcoma, and lymphoma, NLRP3 inhibition suppresses tumor migration, while its activation promotes metastatic spread [42–44]. In pancreatic cancer, it enhances survival via NF-κB signaling and is regulated by PDL1 and CTLA4 [45, 46]. In skin and bone metastases, NLRP3 suppresses NK and T cell responses, favoring tumor progression [44, 47]. Conversely, in CRC liver metastases, HNSCC, and NPC, NLRP3 plays a significant role in promoting immune surveillance, boosting NK cytotoxicity, reshaping the anti-tumor response, and preventing local relapse through IL-1β-driven recruitment of anti-tumor neutrophils [48–50] (Table 1)**.** Here, we aim to provide an overview of inflammasomes' biological and pathological roles that support their potential as promising targets for cancer therapy.

**Table 1:** Inflammation: A key driver of cancer hallmarks

Cancer type	Mechanism	Reference
**Tumor-promoting inflammation**		
Cervical	NLRP3 is implicated in numerous oncogenic stress such as H. pylori, HPV	[38]
Cervical	NLRP3 variants together with polymorphisms influence progression out come	[39]
Prostate	Pathogens are a stimulatory factor for NLRP3 activation in prostate cancer	[2]
**Evading immune destruction**		
Breast	NLRP3 upregulates sphingosine-1-phosphate (S1P) signaling in TAM formation	[40]
Breast	IL-1β suppresses T cell proliferation	[41]
Breast	IL-1β promotes metastasis to bone and lung via the recruitment of γ/δ T cells	[41]
CRC, liver metastasis	Blocking NLRP3 signaling suppresses tumor cell migration	[42]
Fibrosarcoma, lymphoma	Absence of NLRP3 abolishes the immunosurveillance against hyperploid cells	[43]
Multiple cancers	Activation of NLRP3 in TAMs promotes metastatic spread	[40–44]
Pancreas	NLRP3 promotes survival via NF-κB signaling and limited cytotoxic immune cell infiltration	[45]
Pancreas	PDL1 and CTLA4 regulates NLRP3 activation	[46]
Skin	Inhibition of the NLRP3 in TAM suppresses metastasis	[44]
Skin, bone metastasis	NLRP3 suppresses NK and T cell-mediated anti-tumor actions and promotes tumor cell survival	[47]
**Enforcing immune surveillance**		
CRC, liver metastasis	NLRP3 suppresses hepatic metastasis of CRC by promoting NK cytotoxic ability	[48]
HNSCC	NLRP3 reshapes the anti-tumor response through reducing immunosuppressive cells	[49]
NPC	NLRP3 increases IL-1β that inhibits tumor growth and prevents local relapse by recruiting antitumor N1 tumor-associated neutrophils	[50]

### Role of innate and adaptive immunity in cancer

The innate immune system acts as the body's first line of defense, recognizing and eliminating pathogens while activating the adaptive immune system. It consists of physical barriers (such as skin and mucosa), effector cells (including phagocytes, epithelial and endothelial cells, natural killer cells, and innate lymphoid cells), secretions, and pattern recognition receptors (PRRs), including Toll-like receptors [51]. The presence of inflammatory cells within tumors raises a critical question in oncology: How do cancer cells evade immune destruction? Since identifying inflammatory cells in tumors, considerable effort has been devoted to understanding their complex roles in cancer. It is now well established that dysregulation of both innate and adaptive immune responses significantly contributes to tumorigenesis by fostering the selection of aggressive clones, inducing immunosuppression, and promoting cancer cell proliferation and metastasis [52, 53].

Inflammation is a biological response mounted by the body to infections, wounds, and chemical exposure to restore homeostasis and preserve tissue function [54]. The cells and molecules responsible for initiating and coordinating inflammation are critical components of the innate immune system. Tissue-resident macrophages and mast cells are the first responders to harmful stimuli, secreting mediators, including cytokines and chemokines, to recruit other innate immune cells to sites of infection or injury. Neutrophils are among the first cells to respond to these signals, activating and eliminating invading bacteria [55]. If the acute inflammatory response is insufficient to eradicate the insult, macrophages, and T cells are recruited and activated by increased levels of chemokines, growth factors, and cytokines. This process is followed by a resolution phase, during which the local release of signals such as resolvins, protectins, and transforming growth factor β (TGF-β) inhibits further neutrophil recruitment [56]. Instead, monocytes are recruited to the site, differentiate into macrophages, clear dead cells, and initiate tissue repair mechanisms. Chronic inflammation may ensue if the acute inflammatory response fails to resolve correctly [57]. In 1986, Harold F. Dvorak drew a parallel between tumors and "wounds that do not heal," suggesting that tumors can trigger an inflammatory wound-healing response, thus creating favorable conditions for survival and growth [58].

Local inflammation can drive cancer development and progression within the same tissue or organ [59]. For instance, several infections can promote cancer:

1. Helicobacter pylori-induced gastritis can progress to gastric cancer.
2. Chronic hepatitis B or C virus infections can lead to liver cancer.
3. Unresolved infection with human papillomavirus (HPV) can result in cervical cancer.

In addition to the direct carcinogenic effects of these pathogens, the persistent inflammatory environment resulting from failure to clear the infection contributes to cancer development [60, 61]. Chronic inflammatory diseases, even without infections, can also establish a microenvironment primed for tumor development.

The innate immune system comprises various immune cells of myeloid and lymphoid origins and multiple classes of proteins, including cytokines, chemokines, receptors, and complement system components. The activity of these immune elements is not inherently tumor-supportive or tumor-suppressive; instead, their effects depend on the abundance and type of immune cells present in each tissue, the balance of signals within the tumor microenvironment (TME), and the stage of tumor progression. For example, while lung cancer cells engineered to express a potent antigen are rejected by cytotoxic T cells in the lung, similar antigen expression in pancreatic cancer cells can exacerbate the disease, as this site has fewer dendritic cells capable of activating T cells [62]. Additionally, innate immune cells are highly plastic, and their phenotype and function adapt to changes in the local microenvironment. Myeloid cells are heterogeneous, derived from a common progenitor in the bone marrow. They are recruited to tumors and regulate cancer progression, from initiation to invasion and metastasis. The central myeloid cells studied in cancer include macrophages/monocytes, neutrophils, myeloid-derived suppressor cells (MDSCs), and dendritic cells (DCs). Macrophages are large phagocytic cells essential for host defense, especially against bacterial infections, and for maintaining tissue homeostasis by clearing cellular debris and dysfunctional cells. Tissue-resident macrophages originate during embryogenesis, but bone marrow-derived monocytes can also differentiate into macrophages, mainly when recruited in response to injury or infection [63]. Macrophages and their monocyte precursors are often the most abundant immune cell type in solid tumors and are critical contributors to cancer-associated inflammation [64]. Activated inflammatory macrophages release reactive nitrogen species (RNS) and reactive oxygen species (ROS), which have mutagenic potential, as well as cytokines such as tumor necrosis factor α (TNF-α) and interleukins (e.g., IL-1β, IL-6, and IL-12), providing a conducive environment for the development of chronic inflammation-associated cancers. Neutrophils are some of the earliest immune cells to arrive at sites of tissue damage. They combat pathogens through phagocytosis, release of antibacterial proteins and proteases, and formation of neutrophil extracellular traps (NETs) [65]. Factors such as granulocyte-colony stimulating factor (G-CSF) and other cytokines that promote neutrophil differentiation and release from the bone marrow are often elevated in the tumor environment and systemically in cancer patients, resulting in an increased presence of both mature and immature neutrophils [66].

**Fig. 4:**
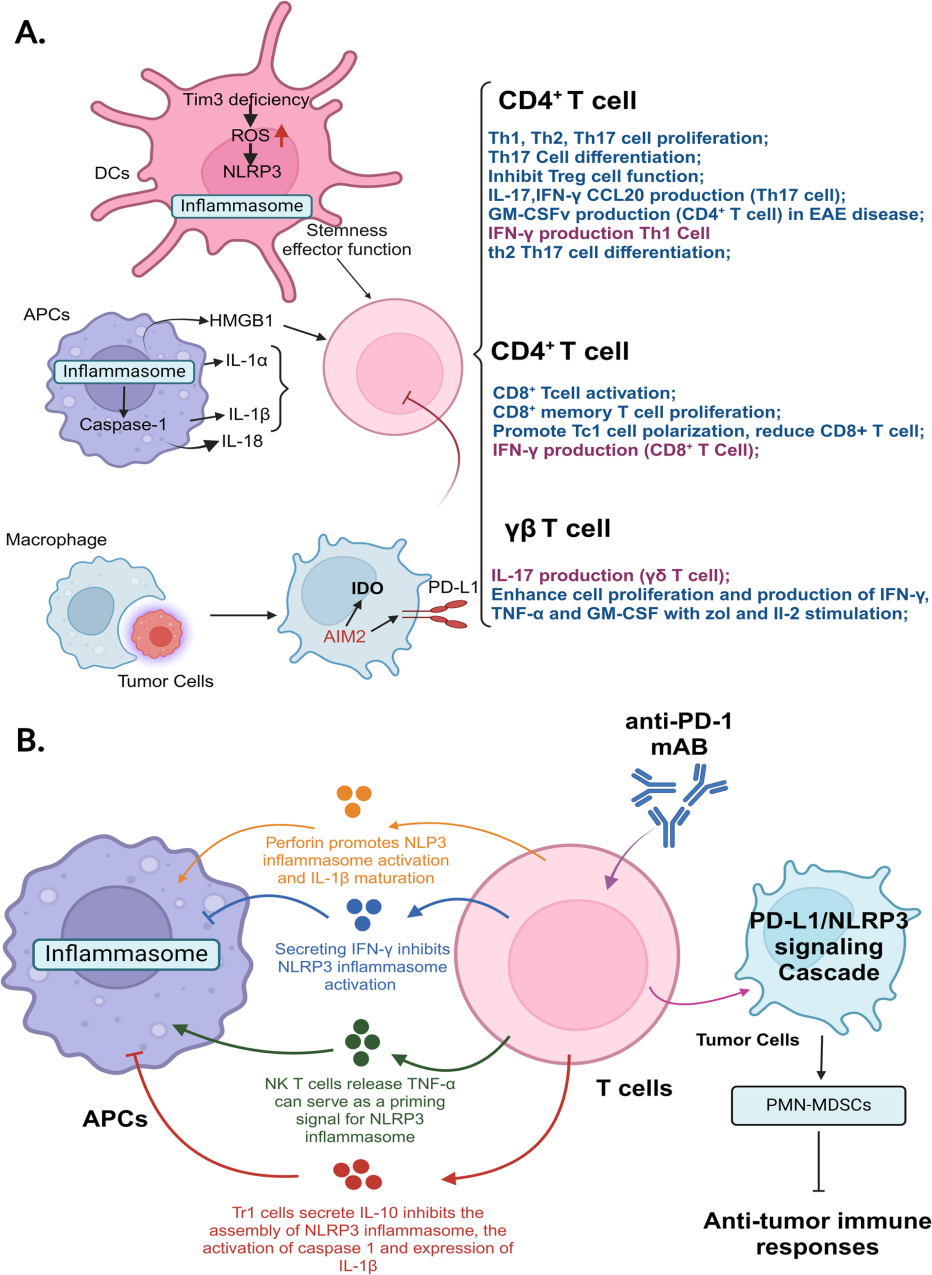
Inflammasomes mediate the dynamic interplay between innate and adaptive immunity, shaping immune responses.** A** Inflammasomes in antigen-presenting cells (APCs) release IL-1 family cytokines and HMGB1, which have a significant impact on T cell immune responses. The arrows in the figure are color-coded to match the font color of the effects they represent. Activation of inflammasomes can also trigger the expression of specific genes in innate immune cells, thereby influencing adaptive immunity. For instance, in macrophages, the AIM2 inflammasome is activated upon recognition of phagocytosed tumor DNA following antibody-dependent cellular phagocytosis (ADCP). This activation leads to the upregulation of IDO and PD-L1, resulting in T cell immunosuppression. Additionally, a deficiency of Tim-3 in dendritic cells (DCs) causes an accumulation of reactive oxygen species (ROS) and subsequent activation of the NLRP3 inflammasome, which enhances the stemness and effector functions of CD8⁺ T cells. **B** Cytokines produced by T cells can modulate the activation or inhibition of inflammasomes in APCs. T cells that secrete perforin and TNF-α can activate the NLRP3 inflammasome, while cytokines like IL-10 and IFN-γ inhibit its assembly. Moreover, the activation of CD8⁺ T cells in response to anti-PD-1 therapy initiates a PD-L1/NLRP3 inflammasome signaling cascade. This cascade ultimately leads to the recruitment of polymorphonuclear myeloid-derived suppressor cells (PMN-MDSCs) into tumor tissues, providing negative feedback that inhibits CD8⁺ T cell activation and anti-tumor immune responses

MDSCs are a heterogeneous population of immature myeloid cells that expand significantly during pathological conditions like cancer. MDSCs are typically categorized into two subgroups: monocytic MDSCs (M-MDSCs) and polymorphonuclear or granulocytic MDSCs (PMN-MDSCs). These cells resemble monocytes and neutrophils morphologically and phenotypically but are characterized by their ability to suppress T cell activity. MDSC expansion is driven by cytokines such as GM-CSF, G-CSF, M-CSF, IL-6, and VEGF, while their immunosuppressive function requires activation by IL-4, IL-13, or TGF-β [67]. DCs are critical players in the innate immune system and serve as a bridge to adaptive immunity. They constantly sample their environment for antigens, danger signals, and pathogens. Upon encountering antigens, DCs migrate to lymphoid tissues (such as the thymus, spleen, and lymph nodes), where they present these antigens to T and B cells through major histocompatibility complex (MHC) class I and II, stimulating antigen-specific immune responses [68]. DCs are traditionally divided into two central populations: conventional DCs (cDCs) and plasmacytoid DCs (pDCs) [69]. Adaptive immunity, mediated by T and B cells, is the body's targeted immune response to specific antigens and can generate long-term immune memory for prolonged protection. T cells differentiate into various subtypes, including CD4 + helper T cells (Th) and CD8 + cytotoxic T cells (CTLs), each with specialized functions. Proper regulation of T cell activation, differentiation, and effector functions is crucial to eliminate infections or tumor cells while avoiding excessive immune reactions or autoimmunity (Fig. 4).

### Fundamental roles of inflammasomes in innate immunity

The immune system is essential for eliminating pathogens, repairing tissue damage, and restoring homeostasis. Inflammasomes are critical components in these innate immune responses. The innate immune system uses germline-encoded pattern recognition receptors (PRRs) to identify microbial threats and damage-associated signals [17]. PRRs are expressed by various cell types, including macrophages, monocytes, dendritic cells, neutrophils, and epithelial cells, which form the first line of defense in the immune system. Acute inflammation is a normal physiological response to infection or tissue injury, and it must be adequately resolved to prevent the onset of chronic inflammation. Chronic unresolved inflammation has been linked to various disorders, including metabolic diseases, autoimmune conditions, and cancer [36]. Research indicates that coordinated resolution mechanisms are initiated shortly after an inflammatory response begins [70]. During this phase, inflammatory cells undergo functional reprogramming to shift from a pro-inflammatory state to a pro-resolving phase [37].

Interestingly, specific molecules involved in the acute inflammatory phase, such as IL-1β, also promote anti-inflammatory responses by inducing the production of IL-10. Inflammasomes play a central role in regulating the expression, maturation, and release of IL-1β, a key regulator of inflammation. At sites of infection or injury, IL-1β promotes the expression of genes involved in inflammation, such as cyclooxygenase-2 (COX-2) and inducible nitric oxide synthase (iNOS), leading to the production of prostaglandin E2 and nitric oxide, respectively. Furthermore, IL-1β enhances the expression of adhesion molecules, including intercellular adhesion molecule-1 (ICAM-1) and vascular cell adhesion molecule-1 (VCAM-1), which facilitate immune cell recruitment to the site of infection or injury. In addition to IL-1β, inflammasomes are also involved in the production of IL-18, another pro-inflammatory cytokine that plays a role in inducing IL-17 expression in Th17 cells and in shaping T cell differentiation towards Th1 or Th2 phenotypes, depending on the context and availability of other cytokines [10]. However, compared to IL-1β, the role of IL-18 in the tumor microenvironment is less defined. Inflammasomes are closely associated with macrophage polarization. Activation of the NLRP3 inflammasome leads to a pro-inflammatory macrophage phenotype characterized by the production of TNF, IL-6, and IL-1β [71, 72].

In contrast, inhibition of NLRP3 signaling promotes the differentiation of microglial cells into an anti-inflammatory phenotype [73]. Macrophages are also essential in cancer initiation and progression. Tumor-associated macrophages (TAMs) comprise a substantial portion of immune cells within the tumor microenvironment (TME), and their heterogeneity affects the efficacy of cancer immunotherapy [64].

Interestingly, a specific population of CD146 + macrophages within the TME has been found to enhance anti-tumor immunity through NLRP3 inflammasome activation [64]. In conventional human dendritic cells (cDCs), the cDC1 subset undergoes cell death following inflammasome activation, whereas the cDC2 subset remains hyperactivated. The cDC2 subset continues to bridge innate and adaptive immunity, particularly in response to inflammasome signals, including NLRP3 activation, which enhances overall immune activity [74, 75]. Neutrophils also play a role in inflammasome activation. During bacterial infections, neutrophils activate inflammasomes such as NLRP3, NLRC4, and NLRP12, becoming a significant source of caspase-1-dependent IL-1β production [76]. Unlike other immune cells, neutrophils do not undergo pyroptosis following canonical inflammasome activation, which might be due to the localization of the N-terminal fragment of gasdermin D (N-GSDMD) in azurophilic granules and LC3 autophagosomes rather than at the plasma membrane [77].

Recent findings suggest that early neutrophil activation involves rapid activation of the NLRP3 inflammasome, which E-selectin triggers. This process entails the transient translocation of GSDMD to the plasma membrane, followed by the release of S100A8/S100A9, and is promptly terminated by ESCRT-III-mediated membrane repair [78]. Additionally, neutrophils secrete IL-1β through distinct, gasdermin-independent mechanisms. Studies involving autophagy-related gene 7 (ATG7)-deficient cells have shown that autophagy is necessary for IL-1β secretion in neutrophils [79]. Furthermore, non-canonical inflammasome activation triggers neutrophil NETosis (gasdermin D-induced cell death) and the release of antimicrobial neutrophil extracellular traps (NETs) through caspase-4/5 in humans and caspase-11 in mice [80]. Recent research also indicates that NLRP3 inflammasome activation in neutrophils promotes NETosis under sterile conditions, such as during the progression of venous thrombosis [81], as shown in Fig. 4A. These findings underscore the significant role of neutrophils in inflammasome activation and highlight the need for further research in this area.

### Inflammasomes role in adaptive immunity

Recent studies have demonstrated that T-cell inflammasome activation is critical in regulating immune responses. In B cells, the NLRP3 inflammasome promotes B cell expansion in adipose tissue, particularly during CpG stimulation. Inhibiting NLRP3-dependent B cell expansion has been shown to reverse metabolic disruptions in aging adipose tissue. Additionally, stimulation of B cells with CpG leads to the activation of NLRP3 and caspase-1, along with the production and secretion of IgM antibodies [82, 83]. In addition to their role in B cells, inflammasomes are also present in T cells, influencing cytokine production and differentiation. Given T-cells' crucial role in adaptive immunity, understanding the mechanisms behind inflammasome activation and regulation within these cells is essential. Initially, inflammasomes were thought to be exclusive to innate immune cells. However, emerging evidence suggests that adaptive immune cells, such as T and B cells, exhibit non-self-recognition behaviors mediated by specific pattern recognition receptor (PRR) systems [84, 85]. These PRRs enhance antigen-driven T-cell responses, trigger effector cytokine release, and activate bystander cells [86].

### Effect of inflammasome activation on T cell function

Inflammasome activation in T cells primarily leads to the maturation and release of pro-inflammatory cytokines, such as IL-1β and IL-18. Once secreted, these cytokines significantly influence T cell activation, proliferation, differentiation, and effector responses through interactions with their receptors, IL-1R and IL-18R. These interactions complement antigen receptor and co-stimulatory signals to enhance the immune response [87]. For instance, in human CD4 + T cells, activation of the NLRP3 inflammasome triggers caspase-1-dependent secretion of IL-1β. This autocrine signaling stimulates the production of IFN-γ and promotes differentiation into Th1 cells, enhancing their pro-inflammatory role in immune responses [88–90]. Thus, inflammasome activation regulates cytokine secretion and primes T-cell differentiation and function, particularly during immune responses to pathogens or tumors.

### Pyroptosis in the regulation of adaptive immunity by inflammasomes

Pyroptosis, a form of programmed cell death, can release large amounts of inflammatory cytokines and potential antigens. The most extensively studied aspect of pyroptosis is its activation through inflammasomes, which promotes the maturation and extracellular release of IL-1β and IL-18. These cytokines modulate adaptive immune functions, including T and B cell responses. This process has been discussed in detail previously. In addition to cytokine release, pyroptosis facilitates the release of small cytoplasmic proteins through gasdermin pores [91], eventually causing cell membrane rupture. This rupture releases large protein complexes such as tetrameric lactate dehydrogenase, lipid mediators, and high mobility group box 1 (HMGB1), a potent pro-inflammatory mediator. The release of these molecules can directly or indirectly influence the function and differentiation of T and B cells. For example, HMGB1 is released extracellularly when immune cells are activated or their membranes are damaged, acting as a pro-inflammatory mediator that promotes an immune response [92]. In LPS-primed macrophages, inflammasome activation triggers caspase activity, facilitating HMGB1 secretion [93]. After cell pyroptosis and membrane rupture, HMGB1 is further released into the extracellular space, contributing to inflammatory signaling [94]. This illustrates how pyroptosis contributes to innate immune responses and influences adaptive immunity by releasing molecules that modulate T and B cell functions.

### Interplay of adaptive and innate immunity

While inflammasome activation in innate immune cells affects adaptive immunity, adaptive immune cells, such as T cells, can also modulate innate immune responses via inflammasome pathways. Specifically, antigen-specific cytotoxic T lymphocytes (CTLs) can activate the NLRP3 inflammasome in antigen-presenting cells (APCs), including dendritic cells and macrophages, through perforin. This results in IL-1β maturation in APCs, creating a positive feedback loop. The secretion of IL-1β by APCs further enhances the priming, activation, and proliferation of CD4 + T cells and CTLs [95]. In the context of immune checkpoint blockade (ICB) therapy, particularly with anti-PD-1 (programmed cell death protein 1) agents, CD8 + T cells that are unblocked by PD-1 inhibition initiate the PD-L1/NLRP3 inflammasome signaling cascade, which recruits granulocytic myeloid-derived suppressor cells (MDSCs) into the tumor microenvironment, thereby enhancing anti-tumor responses [96]. This dynamic interaction between adaptive and innate immunity highlights the bidirectional influence of inflammasome activation in coordinating immune responses, particularly in cancer immunotherapy and immune regulation. Effector and memory CD4 + T cells can suppress inflammasome-mediated caspase-1 activation and IL-1β release in macrophages by downregulating NLRP1 and NLRP3 inflammasomes. For example, in an NLRP3-dependent peritonitis model, effector CD4 + T cells reduce neutrophil recruitment in an antigen-specific manner [97–99]. Additionally, type 1 regulatory T (Tr1) cells, a subset of CD4 + regulatory T cells, inhibit NLRP3 inflammasome activation in macrophages through the secretion of IL-10. Mechanistically, IL-10 promotes mitochondrial autophagy and prevents the accumulation of reactive oxygen species (ROS), inhibiting NLRP3 inflammasome activation. IL-10 also downregulates pro-IL-1β mRNA transcription, caspase-1 activation, and mature IL-1β release in macrophages via the STAT3 signaling pathway [100–103]. Beyond CD4 + and CD8 + T cells, IL-2-stimulated invariant natural killer T (iNKT) cells activate human peripheral blood monocytes through a contact-dependent mechanism, possibly involving interactions with NF-κB signaling receptors in monocytes. This interaction promotes IL-1β transcription and NLRP3 inflammasome-dependent caspase-1 activation, producing IL-1β secretion from monocytes [21]. Furthermore, TNF-α secreted by NKT cells enhances NLRP3 inflammasome activation in APCs, such as dendritic cells, monocytes, and macrophages, leading to the secretion of IL-1β and IL-18 [104]. These findings indicate that adaptive immune cells can modulate innate immune responses through inflammasome activation. However, more research is needed to fully explain the regulatory mechanisms of inflammasomes that bridge innate and adaptive immunity and their role in immune-related diseases (Fig. 4B).

### Inflammasome-targeted therapy

**Fig. 5:**
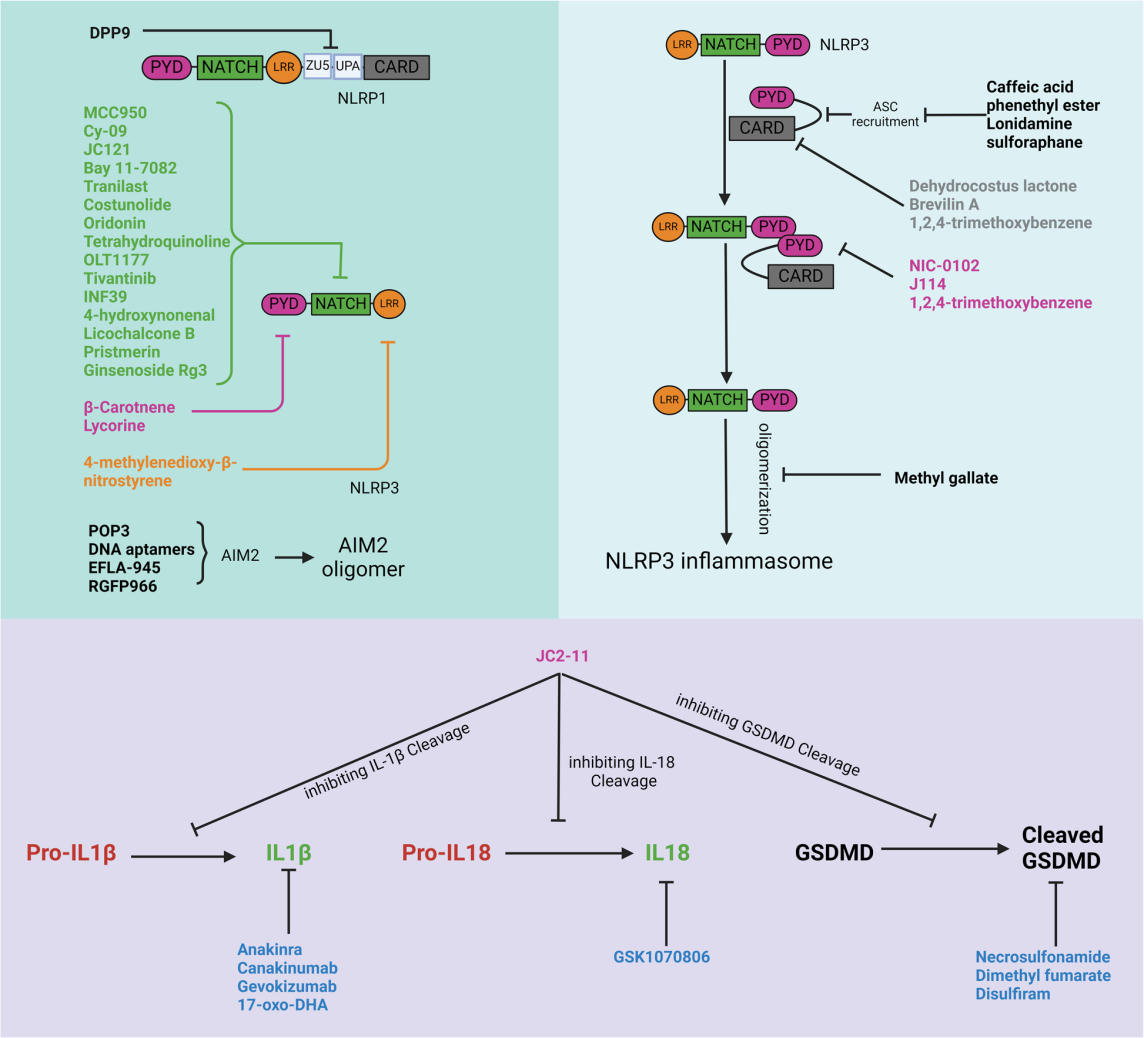
Inhibitors of inflammasomes and related pathways. This figure highlights various inhibitors that specifically target key components of inflammasomes activation, including NLRP1, NLRP3, AIM2, ASC, caspase-1, interleukin-1β, interleukin-18, and GSDMD. These inhibitors by effectively blocking inflammasome activation, suppress pyroptosis and the release of pro-inflammatory cytokines, offering potential therapeutic strategies for inflammation-driven cancer and other diseases

Due to their role in a variety of diseases, inflammasomes have emerged as promising targets for therapeutic intervention. Currently, most FDA-approved drugs act on pathways associated with inflammasome activity rather than directly inhibiting the inflammasomes themselves. However, ongoing research is focused on developing therapies that specifically target inflammasomes, as described in in Fig. 5 and Table 2.

**Table 2:** Overview of current clinical trials targeting inflammation in cancer

Agent	Target	Clinical trial	Drug regimen	Disease and number of participants (*n*)	Phase
Anakinra	IL-1 receptor	NCT04099901	Monotherapy	Multiple myeloma (90)	II
		NCT03430011	Combination with JCARH125	Multiple myeloma (245)	I/II
		NCT04205838	Combination with CAR T cell therapy	Diffuse large B cell lymphoma (36)	II
		NCT04148430	Combination with CAR T cell therapy	B cell acute lymphoblastic leukaemia, B cell lymphoma, B cell non-Hodgkin lymphoma (90)	II
Canakinumab	IL-1β	NCT04239157	Combination with anacitidine	Myelodysplastic syndrome or chronic myelomonocytic leukaemia (60)	II
Isunakinra	IL-1 receptor	NCT04121442	Combination with a PD-(L)1 inhibitor	Solid tumours (25)	I/II
Infliximab	TNF-α	NCT04407247	Combination with vedolizumab	Genitourinary cancer or melanoma (100)	I/II
Infliximab/Certolizumab	TNF-α	NCT03293784	Combination with nivolumab and ipilimumab	Melanoma (30)	I
Tocilizumab	IL-6 receptor	NCT03135171	Combination with trastuzumab and pertuzumab	Breast cancer (20)	I
		NCT04258150	Combination with ipilimumab, nivolumab, and radiotherapy	Pancreatic cancer (30)	II
		NCT02767557	Combination with gemcitabine	Unresectable pancreatic carcinoma (140)	II
		NCT04524871	Combination with atezolizumab and bevacizumab	Advanced-stage liver cancers (100)	I/II
		NCT04338685	Combination with RO7119929	Liver cancer (100)	I
		NCT03821246	Combination with atezolizumab	Prostate adenocarcinoma (34)	II
		NCT04554771	Combination with paclitaxel, carboplatin, and radiotherapy	Oesophageal adenocarcinoma (48)	II
		NCT03970226	Monotherapy	Adamantinomatous craniopharyngioma (27)	I
		NCT03999749	Combination with ipilimumab and nivolumab	Melanoma (67)	II
Siltuximab	IL-6	NCT04191421	Combination with spartalizumab	Metastatic pancreatic adenocarcinoma (42)	I, II
Galunisertib	TGF-β receptor	NCT02452008	Combination with enzalutamide	Prostate cancer (60)	II
		NCT02672475	Combination with paclitaxel	Metastatic androgen receptor-negative triple-negative breast cancer (29)	I
		NCT02688712	Combination with capecitabine and fluorouracil	Rectal adenocarcinoma (50)	II
		NCT03206177	Combination with paclitaxel and carboplatin	Ovarian carcinosarcoma (25)	I
Vactosertib	TGF-β receptor	NCT03724851	Combination with pembrolizumab	Gastrointestinal cancers (67)	I/II
		NCT03732274	Combination with durvalumab	Metastatic non-small-cell lung cancer (63)	I/II
		NCT03698825	Combination with paclitaxel	Metastatic gastric cancer (62)	I/II
		NCT04064190	Combination with durvalumab	Urothelial carcinoma (48)	I/II
		NCT04515979	Combination with pembrolizumab	Non-small-cell lung cancer (55)	II
			Combination with nal-IRI/FL	Pancreas cancer (28)	I
LY3200882	TGF-β receptor	NCT02937272	Monotherapy	Malignant solid tumours (223)	I
NIS793	TGF-β	NCT02947165	Combination with PDR001	Malignant solid tumours (120)	I
AVID200	TGF-β	NCT03834662	Monotherapy	Malignant solid tumours (19)	I
TPX-0022	MET, CSF-1R and SRC	NCT03993873	Monotherapy	Advanced-stage solid tumours (120)	I
IMC-CS4	CSF1R	NCT03153410	Combination with cyclophosphamide, GVAX and pembrolizumab	Pancreatic cancer (12)	I
MCS110	CSF-1	NCT03694977	Combination with PDR001	Gastric cancer (30)	II
Chiauranib	Aurora B, VEGFR, PDGFR, KIT and CSF-1R	NCT03216343	Monotherapy	Small-cell lung cancer (27)	I
Cabiralizumab	CSF-1R	NCT03502330	Combination with APX005M and nivolumab	Advanced-stage melanoma, non-small-cell lung cancer or renal cell carcinoma (120)	I
BLZ945	CSF-1R	NCT02829723	Combination with PDR001	Advanced-stage solid tumours (200)	I/II
DCC-3014	CSF-1R	NCT03069469	Monotherapy	Tenosynovial giant cell tumour (120)	I/II
	CSF-1R	NCT04242238	Combination with avelumab	Advanced-stage arcomas (48)	I
NMS-03592088	FLT3, KIT and CSF-1R	NCT03922100	Monotherapy	Acute myeloid leukaemia or chronic myelomonocytic leukaemia (140)	I/II
Chiauranib	Aurora B, VEGFR, PDGFR, KIT and CSF-1R	NCT03245190	Monotherapy	Hepatocellular carcinoma (35)	I
BMS-813160	CCR2/5	NCT04123379	Combination with nivolumab	Non-small-cell lung cancer or hepatocellular carcinoma (50)	II
		NCT03767582	Combination with nivolumab and GVAX	Pancreatic ductal adenocarcinoma (30)	I/II
		NCT02996110	Combination with nivolumab	Advanced-stage renal cell carcinoma (200)	II
		NCT03496662	Combination with nivolumab, gemcitabine and paclitaxel	Pancreatic ductal adenocarcinoma (53)	I/II
Leronlimab	CCR5	NCT04504942	Monotherapy	Advanced-stage solid tumours (30)	II
		NCT03838367	Combination with carboplatin	Triple-negative breast neoplasms (48)	I/II
SX-682	CXCR1/2	NCT03161431	Combination with pembrolizumab	Metastatic melanoma (77)	I
AZD5069	CXCR2	NCT03177187	Combination with enzalutamide	Metastatic castration-resistant prostate cancer (49)	I/II
Plerixafor	CXCR4	NCT02605460	Combination with busulfan and cyclophosphamide	Acute myeloid leukaemia or acute lymphoid leukaemia (20)	II
BL-8040	CXCR4	NCT02763384	Combination with nelarabine	T cell acute lymphoblastic leukaemia (20)	II
IPH5401	C5aR	NCT03665129	Combination with durvalumab	Advanced-stage solid tumours (140)	I

### Sensor protein modulation

Considerable efforts have been made in recent years to develop inhibitors that target inflammasome sensor proteins as potential treatments for inflammatory diseases. For example, cytosolic DPP9 binds to the C-terminus of NLRP1, thereby preventing inflammasome activation [105, 106]. MCC950, an inhibitor of NLRP3, blocks activation by targeting its NACHT domain, explicitly inhibiting the Walker B motif and preventing ATP hydrolysis [107]. Tranilast, commonly used for allergies, disrupts NLRP3 inflammasome assembly by directly binding to its NACHT domain, inhibiting interactions with ASC (Zhuang et al., 2020). Other drugs, such as CY-09, act on the Walker A motif of NLRP3, displacing ATP without affecting NLRP1 or NLRC4 [108]. Analog compounds, such as N-benzyl 5-(4-sulfamoylbenzylidene-2-thioxothiazolidin-4-one), have been shown to selectively inhibit the interaction between NLRP3 and ASC, reducing inflammasome assembly [109]. Oridonin forms a covalent bond with Cys279 in the NACHT domain of NLRP3, inhibiting its interaction with NEK7 and thus reducing activation. Tetrahydroquinoline also inhibits NLRP3 inflammasome activation by binding to its NACHT domain and preventing ASC oligomerization [110]. Another compound, OLT1177, binds directly to NLRP3, inhibiting ATPase activity, caspase-1 activation, and IL-1β production in cells from patients with cryopyrin-associated periodic syndrome [111]. It showed good safety and tolerability in a Phase I trial involving patients with heart failure [112]. Other compounds, such as β-carotene, also inhibit NLRP3 inflammasome activation by binding to its PYD domain, thereby decreasing macrophage activation [113]. 4-Methylenedioxy-β-nitrostyrene inhibits NLRP3 by targeting the NACHT and LRR domains, reducing ATPase activity [114]. Lycorine binds to residues of the PYD domain, interfering with the interaction between NLRP3 and ASC [115]. Tivantinib, an anticancer agent, also inhibits the ATPase activity of NLRP3 and its assembly [116]. Bay 11–7082 inhibits inflammasome activation by alkylating cysteines in the ATPase region of NLRP3, thereby disrupting ASC pyroptosome formation [117]. However, it also inhibits IKKβ kinase activity, modulating NLRP3 expression through the NF-κB pathway [118]. Additionally, 4-hydroxynonenal disrupts the NLRP3-NEK7 interaction [119]. NIC-0102, a proteasome inhibitor, promotes polyubiquitination of NLRP3, disrupting its interaction with ASC and thereby inhibiting inflammasome activation [120]. INF39 also inhibits the interaction between NEK7 and NLRP3, blocking subsequent interactions and ASC oligomerization [121].

### Inhibitors of AIM2 and NLRP3 inflammasomes

The PYD-only protein POP3 inhibits AIM2 inflammasomes by competing with ASC for recruitment (7462). Obovatol, a bisphenol compound, inhibits AIM2 and NLRP3 inflammasomes by preventing ASC pyroptosome formation [122]. RGFP966, an inhibitor of histone deacetylase 3, regulates AIM2 inflammasome activity temporally [71]. DNA aptamers have also shown promise inhibiting AIM2 inflammasome activity [123]. Roxadustat has been found to inhibit AIM2 in a CD73-dependent manner [124]. Several natural bioactive compounds also exhibit potential for inhibiting NLRP3 and AIM2 inflammasomes. For instance, costunolide, a primary active ingredient in the medicinal herb Saussurea lappa, covalently binds to Cys598 in the NACHT domain of NLRP3, inhibiting ATPase activity [125]. EFLA-945, an extract from red grapevine leaves, prevents DNA from entering macrophages, thereby reducing AIM2 inflammasome activation [126]. Ethanolic extracts from the seeds of Cornus officinalis inhibit AIM2 speck formation induced by double-stranded DNA [127].

### ASC modulation

ASC is the adaptor protein in canonical inflammasomes, making it a viable target for regulating inflammasome activity. J114 inhibits the interaction between NLRP3/AIM2 and ASC, reducing ASC oligomerization [128]. Caffeic acid phenethyl ester (CAPE) binds directly to ASC and disrupts NLRP3-ASC interactions [129]. In mouse models of gouty arthritis, CAPE administration decreased caspase-1 activation and IL-1β release. Additionally, VHHASC, an alpaca-derived single-domain antibody, impairs ASC-CARD interactions while maintaining ASC's PYD function [130]. Lonidamine, a glycolysis inhibitor, inhibits ASC oligomerization by directly binding to it [131]. Dehydrocostus lactone, also from Saussurea lappa, inhibits ASC oligomerization [132]. Sulforaphane might inhibit NLRP3 inflammasome activation by affecting ASC or caspase-1 [80]. Brevilin A, derived from Centipeda minima, reduces NLRP3 inflammasome activation by blocking ASC oligomerization [133]. Additionally, 1,2,4-trimethoxybenzene, found in essential oils, inhibits ASC oligomerization and NLRP3-ASC interactions [134].

### Caspase modulation

Caspase-1 inhibitors have become a key focus in the pharmaceutical industry, as caspase-1 is central to all canonical inflammasomes. VX-765 (belnacasan) selectively inhibits caspase-1 by modifying its catalytic cysteine residues and has shown potential in reducing inflammation [135]. It has also been shown to improve cognitive impairments in Alzheimer's models and preserve cardiac function in myocardial infarction, though long-term use led to hepatotoxicity in animal studies. Sennoside A, commonly used in dietary supplements, inhibits caspase-1 activity via the P2X7 pathway, reducing inflammation mediated by NLRP3 and AIM2 [132]. Pralnacasan, an orally available non-peptide compound, inhibits caspase-1, reducing joint damage in osteoarthritis models and mitigating colitis in mice [136, 137].

### IL-1/IL-18 modulators

IL-1β and IL-18 are the primary pro-inflammatory cytokines activated by inflammasomes, playing critical roles in various diseases. Anakinra, a recombinant IL-1 receptor antagonist, reduces disease activity in rheumatoid arthritis (RA) patients and improves inflammatory and glycemic parameters in RA and type 2 diabetes [138]. Canakinumab, an IL-1β monoclonal antibody, is approved for treating cryopyrin-associated periodic syndromes and has been shown to reduce cardiovascular event recurrence [139, 140]. Rilonacept, another IL-1 inhibitor, has been approved for Phase 3 trials in recurrent pericarditis [141, 142]. Gevokizumab, a unique IL-1β-binding protein, is being evaluated for inflammatory diseases [143]. IL-18 blockers are also being investigated. GSK1070806, a recombinant IL-18-neutralizing antibody, showed promising results in a Phase 1 trial involving healthy and obese males [144, 145].

### GSDMD modulators

Gasdermin D (GSDMD) is essential for pyroptosis, making it a significant target for treating inflammasome-related diseases. Disulfiram, an FDA-approved drug for alcoholism, inhibits GSDMD pore formation by modifying cysteine residues [146]. In mice, disulfiram reduced pyroptosis, cytokine release, and LPS-induced septic death. Necrosulfonamide and dimethyl fumarate also prevent GSDMD clustering, inhibiting pyroptosis in models of Alzheimer's disease [147]. Further research is needed to explore the potential of GSDMD inhibitors for treating inflammasome-related conditions.

## Conclusion

Inflammasomes, particularly the NLRP3 inflammasome, play complex and dual roles in cancer immunity. Their activation within the tumor microenvironment influences innate immune responses and shapes adaptive immunity by modulating cytokine secretion, immune cell recruitment, and T-cell differentiation. While chronic inflammasome activation can promote tumor initiation and progression through sustained inflammation, it also presents opportunities for novel therapeutic interventions aimed at enhancing anti-tumor immunity. Recent advances have highlighted the potential of targeting inflammasome components such as sensor proteins (e.g., NLRP3, AIM2), adaptor proteins (e.g., ASC), caspase-1, and downstream cytokines IL-1β and IL-18—to modulate the immune response against cancer. Various inhibitors and modulators are under investigation, some have shown promise in preclinical studies and are currently undergoing clinical evaluation. These therapeutic strategies aim to suppress the pro-tumorigenic effects of chronic inflammation while enhancing the immune system's ability to recognize and eliminate cancer cells.

### Future perspectives

Future studies should prioritize elucidating the mechanisms through which inflammasomes mediates their dual roles in tumor progression and suppression. to tumor-promoting and tumor-suppressing activities. A deeper understanding of the context-dependent roles of inflammasomes across diverse cancer types and stages could lead to the development of more precise, personalized, and effective therapeutic strategies. Additionally, investigating the interplay between inflammasome activation and other immune pathways, such as immune checkpoint blockade therapies, may uncover synergistic strategies to overcome immune resistance in tumors. Moreover, developing selective and potent inflammasome inhibitors with minimal off-target effects remains a significant challenge. Advances in drug design and delivery methods could improve these potential therapies' efficacy and safety profiles. Exploring natural compounds and bioactive agents from medicinal plants may offer alternative avenues for modulation of inflammasome. In conclusion, targeting inflammasomes represents a promising strategy to bridge innate and adaptive immune responses in cancer therapy. By harnessing the dual roles of inflammasomes, future interventions may effectively suppress tumor-promoting inflammation while activating robust anti-tumor immunity, ultimately improving clinical outcomes for cancer patients.

## Data Availability

No datasets were generated or analysed during the current study.
